# Francis Crick, cross-worlds influencer: A narrative model to historicize big bioscience

**DOI:** 10.1016/j.shpsc.2015.08.003

**Published:** 2016-02

**Authors:** Christine Aicardi

**Affiliations:** Department of Social Science, Health and Medicine, King's College London, Strand, London WC2R 2LS, United Kingdom

**Keywords:** Francis Crick, Historiography, Interdisciplinarity, Influence, Brokerage, Big science

## Abstract

The essay is an empirical case study of famed British scientist Francis Crick. Viewing him as a ‘cross-worlds influencer’ who was moreover dedicated to a cause, I have tried to understand how these two characteristics influenced the trajectory of his long career and how they shaped his contributions to the diverse research fields in which he was active, and concluded that these characteristics reconfigure Crick's career into a coherent whole. First, I identify a major thread running through Crick's career: helping organise ‘un-disciplined’ new research fields, and show that his successive choices were not serendipitous but motivated by what he construed as a crusade against ‘vitalism’: anti-vitalism was a defining driver of his career. I then examine how Crick put his skills as a crossworlds influencer to the service of his cause, by helping organise his chosen fields of intervention. I argue that his activities as a cross-worlds influencer were an integral part of his way of ‘doing science’ and that his contributions to science, neuroscience in particular, should be re-evaluated in this light. This leads me to advance a possible strategy for historians to investigate big bioscience fields. Following Abir-Am, I propose to trace their genealogies back to the fluctuating semi-institutional gatherings and the institutional structures that sustained them. My research on Crick supports the view that such studies can bring insights into the question of why the contours of contemporary big bioscience endeavours have come to be shaped the way they are. Further, the essay provides a heuristic device for approaching these enquiries: ‘follow the cross-worlds influencers’ who worked to build and organise these semi-institutional gatherings and institutional structures.

## Highlights

• Historians should look for alternatives to big data for navigating contemporary big bioscience.
• Broadening the scope of what ‘doing science’ encompasses may bring new insights.
• ‘Follow the cross-worlds influencers’ is a heuristic device for historicizing big bioscience.
• Francis Crick was a cross-world influencer dedicated to promoting an ‘anti-vitalist’ worldview.
• His contributions to science, neuroscience in particular, should be re-evaluated in this light.

When citing this paper, please use the full journal title *Studies in History and Philosophy of Biological and Biomedical Sciences*

## 1 Introduction

The unprecedented rise of large-scale, collaborative, multi-disciplinary ‘Big Science’ projects, in the wake of World War II, poses new challenges for archivists and historians of science. How to document them and write their history is much debated. One prominent difficulty is the large number and diversity of the participants involved, and the huge mass of data they produce. In our knowledge economies, humanities are encouraged to follow the growing trend towards ‘Big Data’ and develop tools and methods to exploit the vast volumes of data that ‘Big Science’ and information society generate. In history of science as in many other domains, data-driven research appears as a promising response to the challenges of the ‘data deluge’ caused by the overabundance of sources.^1^ However, this approach does not go unchallenged (Scheinfeldt, 2012). Indeed, one overarching aim of the collection to which this essay belongs, is to propose other possible strategies to find a way into, and make sense of, the data-crowded labyrinths of the contemporary biosciences.

In his contribution, Miguel García-Sancho argues that the main issue is not so much the proliferation of sources, as a lack of suitable narrative models (García-Sancho, 2016). This view is consistent with that of skeptics in other research fields who view the increasingly popular strategy of developing tools and methods for data-driven research as a substitute for theory-driven research, or, as the most radical would have it, as a substitute for thinking (Carandini, 2015, de Chadarevian, 2009, Fisher, 2015). Based on his experience researching the history of genomics, García-Sancho proposes to use the administrative archives of big science projects as alternatives to individual scientists' papers and to follow “the synthetic voice of the invisible administrator.” He justifies this approach on the grounds that “[t]he brokering expertise of big science administrators, navigating among the many actors involved in the projects and harmonising their conflicting views, constitutes a privileged point of entrance into the rhizome of genomics.” (García-Sancho, 2016).

This argument calls for two observations. First, García-Sancho's emphasis on the brokering expertise of big science administrators implies that the value of administrators as entry points into big science projects depends not so much on their administrative management skills as on their aptitude at ‘managing by influence’ across different social worlds. Influence has been the object of much attention in the business and management literature, especially in relation to leadership. Although much of this work has been published in the grey literature aimed at managers and top executives, political communicators, social marketers and the like,^2^ it has also found its way into more scholarly publications.^3^ Here, influence is commonly defined as the capacity to affect others' ideas, opinions and actions by intangible or indirect means, instead of through direct authority. Influence, it is commonly argued, is particularly important for roles of leadership in environments where the focus in on strategy and where the best way to accomplish objectives is through collaboration and persuasion, rather than directive, bureaucratic management. Major factors in influence include commitment to a vision, consistency in message, and other people's respect for and liking of the influencer. Of paramount importance is the ability to build and nurture interpersonal trust relationships, as a platform for influence (Cialdini, 2007, Kaufman, 2011).

In his argument, García-Sancho does not disentangle the distinct roles of the two forms of management, administration and influence, in the organisation of interdisciplinary, collaborative science. This is characteristic of much history and sociology of science. There have been few attempts to examine how the capacity to exert influence across different contexts, what I will term ‘cross-worlds influencing,’ contributes to the makeup of science, to the directions in which science develops, its organisation and practices. Still, there are noteworthy exceptions. The overarching goal of the collective volume *The Brokered World. Go-Betweens and Global Intelligence, 1770–1820* has been to bring to light “the largely ignored role of go-betweens in the very construction of [the] modern world, notably in the domain of knowledge and sciences.” (Schaffer, Roberts, Raj, & Delbourgo, 2009) More relevant to our concern with contemporary big science, Shapin's study of the post-World War II scientist as institution builder and entrepreneur has shown that, in large collaborative and interdisciplinary projects, “the personal, the familiar, and even the charismatic” are ever more important to research management; that vision, inspiration, stimulation, encouragement, capacity to relate to various individualities and diverse research interests—all qualities that map onto the key components of influential leadership highlighted above—were far more importance for the flourishing of such research projects than well-oiled bureaucratic task management (Shapin, 2008, chapter 6 especially).

The sociology of science has paid some attention to skills relevant to cross-worlds influencing in relation to multidisciplinary big science. I have particularly in mind Harry Collins and Robert Evans' elaboration of the concept of ‘interactional expertise’ in their Studies of Experience and Expertise programme.^4^ ‘Interactional expertise’ was initially conceived in the context of ethnographies of science and it was defined as “enough expertise to interact interestingly with participants and carry out a sociological analysis” as opposed to ‘no expertise’ (“insufficient to conduct a sociological analysis or do quasi-participatory fieldwork”) and ‘contributory expertise’ (“enough expertise to contribute to the science of the field being analysed”) (Collins & Evans, 2002, p. 254). The concept was subsequently generalized as “the product of a successful linguistic socialization. Although expressed as language alone, it cannot be too heavily stressed, interactional expertise is tacit knowledge-laden and context specific.” (Collins, Evans, & Gorman, 2007, p. 661). In the Studies of Expertise and Experience programme, Collins has qualified his conception of language as ‘practice language,’ whose defining feature is “its substantive (often tacit) content,” and has gone on to reconfigure ‘contributory experts’ as a subset of the class of ‘interactional experts’, further blurring the roles of language and practice in the definition of interactional expertise (Collins, 2011, pp. 274–276). In its extended version, ‘interactional expertise’ has been recognized as essential to the coordination of activities in a complex division of labour (Collins, 2011, p. 284) and it has been presented as a linchpin of ‘fractionated trading zones’, characterized as interdisciplinary partnership that “involves fractions of cultures as the medium of interchange … which are mediated by language largely in the absence of the material” (Collins et al., 2007, p. 660). Multidisciplinary and collaborative ‘big science’ fields are typical instances of such partnerships.^5^ Coming back to my concern with cross-worlds influencing, interactional expertise—mastery of the tacit knowledge pertaining to a domain of expertise—is a major asset when engaging with the corresponding specialist community. In the case of big science projects, an individual who is able to develop interactional expertise in several of the research fields involved is in a position to play a privileged role brokering and building trust between distinct research groups, thus influencing the shape and direction of the collaboration. Coming from the sociology of social networks tradition, Ronald Burt reaches similar conclusions, arguing that ‘between-group brokers’ who exploit the ‘structural holes’ in a social network (i.e. brokers lying on weaker connections between densely clustered groups within the network) play a special role in generating social capital (Burt, 2001, Burt, 2002, Burt, 2004). On these premises, I will propose that beside project administrators, a wider and more diverse array of ‘cross-worlds influencers’ are worth pursuing as privileged ‘entry points’ into big science.

My second observation on García-Sancho's argument is that ‘the rhizome’ of genomics is not free-floating. To pursue the botanical metaphor, this complex outgrowth has a specific genealogy in which only certain disciplinary lineages are represented, and has grown out of a specific terrain. In the early 1990s, Pnina Abir-Am suggested that historians should focus on “intermediary units of sociohistorical analysis … collaborative patterns of various duration, such as research schools, circles, clubs, and other informal gatherings, which combine a definable social structure with coordinated research programs”, on the grounds that they were indispensable for understanding the history of many interdisciplinary fields in twentieth-century science (Abir-Am, 1991, p. 342). If we follow Abir-Am, the broad category of intermediary analytical units, of fluctuating collaborative patterns, that she was bringing to attention were instrumental in creating the sheltered spaces into which many a 20th-century interdisciplinary research programme took root, eventually growing into fully-fledged endeavours in ‘big science’. These gatherings thus deserve attention for the ways in which they have ‘landscaped’ the grounds and prepared the terrain, where specific hybrids (genomics being one of several) took root and grew while others did not.

A characteristic common to all Abir-Am's examples of gatherings is that they are ‘semi-institutional,’ in the sense that they sit in-between the public and private spheres: grass-root, co-opted and often confidential on the one hand; endorsed and supported by institutions on the other.^6^ I would argue that their semi-institutional nature is a salient quality of such gatherings, essential to the role they have played in the development of 20th-century life sciences. They were sites where the interpersonal trust relationships required by interdisciplinary collaboration could grow and deepen in a framework shaped by institutionally-sanctioned scientific aims. In this essay, my point is that semi-institutional gatherings provide privileged playing fields for cross-worlds influencers, offering environments whose informality discourages directive authority and encourages the building of trust.

The essay is an empirical case study of famed British scientist Francis Crick. Viewing him as a cross-worlds influencer who was moreover dedicated to a cause, I have tried to understand how these two characteristics influenced the trajectory of his long career and how they shaped his contributions to the diverse research fields in which he was active: I have reached the conclusion that these characteristics reconfigure Crick's career into a coherent whole. First, I identify a major thread running through Crick's career: helping organise ‘un-disciplined’ new research fields, and show that his successive choices were not serendipitous but motivated by what he construed as a crusade against ‘vitalism’: anti-vitalism was a defining driver of his career. I then examine how Crick put his skills as a cross-worlds influencer to the service of his cause, by helping organise his chosen fields of intervention. I argue that his activities as a cross-worlds influencer were an integral part of his way of ‘doing science’ and that his contributions to science, neuroscience in particular, should be re-evaluated in this light.

This leads me to advance a possible strategy for historians to investigate big science. Following Abir-Am, I propose to trace their genealogies back to the fluctuating semi-institutional gatherings and the institutional structures that sustained them. My research on Crick supports the view that such studies can bring insights into the question of why the contours of contemporary big science projects have come to be shaped the way they are.^7^ Further, the essay provides a heuristic device for approaching these enquiries: follow the cross-worlds influencers who worked to build and organise these semi-institutional gatherings and institutional structures.

## 2 A self-constructed ‘anti-vitalist’

In the course of his scientific career, Francis Crick changed research fields several times, from protein crystallography, molecular genetics, developmental cell biology and the chemical origin of life while at the UK Medical Research Council Laboratory of Molecular Biology in Cambridge (LMB), to the neuroscience of vision and the science of consciousness while at the Salk Institute for Biological Studies in La Jolla, California. This represents not quite thirty years researching the broad domain of molecular biology and genetics at the LMB, for over twenty-five years doing neuroscience at the Salk Institute.

Mapping the detailed chronology of Crick's scientific activities (Olby, 2009) onto the wider developments of post-World War II life sciences shows that a defining element of his scientific career was that he repeatedly engaged with research fields when they were still fledgling scientific pursuits jostling at the margins of established disciplines. He was frank about this. What he was interested in was to build general theoretical frameworks that would foster experimental research programmes. And he wanted to do so in areas of biology that lacked such frameworks because they revolved around questions that “seemed beyond the power of science to explain.” (Crick, 1988, p. 17). Once he deemed that a field was organized enough, scientifically speaking, he would feel the urge to turn to another, in need of the lights of science:

“… by 1966, we realized that the *foundations* of molecular biology were now sufficiently firmly outlined that they could be used as a fairly secure basis for the prolonged task of filling in the many details. Sydney Brenner^8^ and I thought that it was time to move on to new fields.” (Crick, 1988, p. 144)

By 1966, molecular biology was a far cry from its state in 1947 when Crick had applied to the UK Medical Research Council to pursue his postgraduate studies in protein crystallography at the brand new MRC Unit for the Study of the Molecular Structure of Biological Systems in Cambridge—the future LMB. At the time, the unit was housed in the Cavendish Laboratory and counted just two full time researchers, Max Perutz and John Kendrew (de Chadarevian, 2002, p. 65); and their research field, which was not yet called ‘molecular biology’, was predominantly practised by defectors from physics and chemistry and still considered by traditionalists in the physics and life sciences departments as an upstart challenger of doubtful promise (for instance, Abir-Am, 1987, de Chadarevian, 2002, Kay, 1993). This early set-up was a far cry from the LMB that was to grow out of the MRC Unit, which, in May 2015, counted around fifty research groups.^9^ Robert Olby's richly detailed biography of Crick and Soraya de Chadarevian's exceptionally well documented history of the LMB and of post-World War II British molecular biology have amply demonstrated that Crick actively helped instituting molecular biology into a powerful scientific discipline (de Chadarevian, 2002, Olby, 2009). At the LMB, until 1976 and his move to California, Crick devoted his energies to helping molecular biology off the ground, consolidating its institutional foundations, and strengthening its research community. He also worked assiduously to expand its remit to genetics, cell and developmental biology, and neurobiology. The sustained diversification that Crick and his close collaborator Sydney Brenner engaged in was remarkable in its breadth, and as de Chadarevian has pointed out: “[w]hat is surprising is how unproblematic the expansion of research interest seems to have been.” (de Chadarevian, 2002, p. 197).

Retrospectively, it is hard to believe that the burgeoning research field which Crick joined in 1947 could have been considered a flaky pursuit. Yet this is how Brenner recently reminisced about the era:

“To have seen the development of a subject, which was looked upon with disdain by the establishment from the very start, actually become the basis of our whole approach to biology today. That is something that was worth living for. … What people don't realise is that at the beginning, it was just a handful of people who saw the light, if I can put it that way. So it was like belonging to an evangelical sect, because there were so few of us, and all the others sort of thought that there was something wrong with us. They weren't willing to believe. … I remember when going to London to talk at meetings, people used to ask me what am I going to do in London, and I used to tell them I'm going to preach to the heathens.”(Dzeng, 2014)

Confrontation with the establishment, misunderstood genius, are common tropes of scientists' memories. Still, if we trust de Chadarevian, it is clear for example that even in the late 1950s, geneticists had yet to develop a general interest in molecular genetics; and her account of the long and difficult negotiations over the relocation the expanding lab, between the MRC Unit research group, the University of Cambridge and the Medical Research Council, which lasted from 1953 until the move into the new LMB buildings in 1962, shows that a research group “which showed no loyalty to a particular discipline” was a thorny issue for the university. The latter resisted the researchers' plans and offered little cooperation (de Chadarevian, 2002).

Another research field in which Crick became involved when it was still in an embryonic state was the origins of life. For a period of time between the late 1960s and early 1970s, he collaborated with theoretical chemist Leslie Orgel, to evaluate whether it was possible to attack the question scientifically. Although their joint inquiry remained (by their own admission) at the speculative level^10^ and their foray into ‘directed panspermia’^11^ attracted a fair share of sarcasm from the scientific establishment (Crick, 1981, Crick and Orgel, 1973, Crick and Orgel, 1993, Olby, 2009, pp. 369–362; Ridley, 2006, pp. 171–174), Orgel went on to pursue for over thirty years a solid research programme in exobiology and the chemical evolution of life, largely funded by NASA.

In 1976, Crick moved to California to join the Salk Institute and to enter the field of neuroscience. He first chose to focus on the neuroscience of vision, when according to visual psychophysicist V.S. Ramachandran, ‘‘Vision seems to be in a state of confusion similar to pre-DNA molecular biology.”^12^ Then, from the late 1980s onward, he turned to the neuroscience of consciousness, at a time when consciousness studies were still seen as downright unscientific. Again, Crick invested himself in organising these new fields and making them scientifically respectable. Describing Crick's role in transforming the San Diego area into a hub of neuroscientific research, Ramachandran has used a vivid botanical metaphor:

“… he was instrumental in having many of us in neuroscience and psychology move to La Jolla in the early eighties —transforming it into “neuron valley” (He had the foresight to bring Terry Sejnowski and the Churchlands to UCSD at a time when the kinds of topics they worked on were not considered especially fashionable). We all thought of him as a great Sequoia tree under whose branches many of us saplings eked out a precarious living.” (Ramachandran, 2004)^13^

A glaring question at this point is whether Crick's successive choices of research field were serendipitous or if there was a common thread uniting them beyond their fledgling state. According to his autobiography, what they had in common was that they passed the ‘gossip test’, i.e. “… what you are interested in is what you gossip about,” and they tackled problems which were deemed by most, under what Crick assumed to be the influence of religious dogma, as “… beyond the power of science to explain” (Crick, 1981, pp. 16–17). *Of Molecules and Men*, a small and rather obscure book derived from the John Danz Lectures that Crick was invited to give at the University of Washington in February and March 1966, provides a more insightful answer. This series of three lectures, a much expanded version of the lecture on ‘The Molecular Basis of Life’ that Crick had given to the Cambridge Humanists in 1963, was entitled ‘Is vitalism dead?’ The resulting book was literally a manifesto against vitalism (Crick, 1966; pp. ix–x; Olby, 2009, p. 325).

Crick was not attempting to develop a deep philosophical critique of vitalism. Instead, he understood the term in a general way, as implying “some special force directing the growth or the behaviour of living systems which cannot be understood by our ordinary notions of physics and chemistry” (Crick, 1966, p. 16). According to Crick, vitalism, a doctrine used to explain away complicated biological phenomena that could not be easily explained, was not dead. He thought that in fact, in certain areas of science, vitalism was enjoying a resurgence, of which the book offered examples. For him, it was a remnant of the moribund “old, or literary culture, which was based originally on Christian values” (Crick, 1966, p. 93) and which science was in the process of uprooting: exact scientifically acquired knowledge was the enemy of vitalism. Crick went on to examine the three areas of biology where, in his opinion, vitalist ideas were still lurking. They turned out to be precisely the same three broad domains that he was to research in the course of his career: molecular biology, the origin of life, and the higher nervous system. He considered the latter to be the most scientifically backward: “Here vitalistic ideas not only are commonplace among educated laymen, but are held by several of the leading workers in this field.”(Crick, 1966, p. 98)

The idea that the struggle against vitalism was the overarching thread running through Crick's life is supported by a speech given by his son Michael at the Francis Crick Memorial service held in September 2004 at the Salk Institute:

“… what made Francis Crick tick? … Obviously something very powerful was driving him, but what was it? … My thesis here today is that Francis' driving quest was to knock the final nails in the coffin of vitalism. … [Francis] wanted to put these ideas to bed, first of all with the structure of DNA, and then he moved on to how proteins were synthesized, and then he moved on to cell biology and how animals grew and so forth. But beside what Francis called ‘the frontier between the living and the non-living’, he thought that there were two other pillars that had to be demolished in order for vitalism to be finally dead. One was the origin of life … And Leslie Orgel has been working on that for the last 30 years … So once Francis deemed that the work achieved in molecular biology had freed the question of ‘what is life’ from vitalism, and that the question of the origin of life was in good hands, he then moved on to attack the last pillar that had to be demolished and that was consciousness … Francis was a man who was trying his whole life to win an argument. I never understood who he was arguing against but he had this total conviction that he had to win this argument.”^14^

Crick's crusader's tendencies did not disappear when he moved on to neuroscience. Ralph Siegel and Ed Callaway, two neuroscientists of a younger generation who enjoyed Crick's support, described him in their obituary as an “evangelical atheist” who “… was building an army to help him take on consciousness.” (Siegel & Callaway, 2004).

Olby, Crick's official biographer, has also sought to understand “why Crick was so passionate, and at times, vehement, about the new knowledge of molecular biology” and why he “assumed the role of preacher and evangelist.” (Olby, 2009, Preface) Olby's historiographical approach led him to look for intellectual explanations at the level of the autonomous individual, viewing Crick as a far-sighted scientist rationally responding to opposition from other scientists and understanding the potential benefits of this new science for society (Olby, 2009, Preface). I have adopted a more sociological perspective, approaching Crick as a social agent best understood through the roles he played across the social groups he belonged to; and I have rejected the clean separation between reason and beliefs, embracing the idea that “[v]isions, images and beliefs cannot sharply be demarcated from knowledge. Knowing is inevitably rooted in some set of beliefs.” (Nowotny, Scott, & Gibbons, 2001, p. 232)

The aim of the historian is to try and understand the past on her subjects' own terms. In the case of science, this means projecting herself back into the ‘now’ that scientists experienced as their work unfolded over time. In the early stages of a research programme especially, this implies thinking of science-in-the-making as still predominantly a matter of beliefs, belonging to the realm of science-as-it-could-be. Indeed, Crick himself occasionally let slip that his scientific certainties amounted to a belief system. Writing about “the borderline between the living and the nonliving”—the subject matter of molecular biology—he recalled that “[n]ot so many years ago this seemed infinitely mysterious, and only by a very considerable act of faith could one believe that an explanation would be possible in terms of physics and chemistry.” (Crick, 1966, pp. 16–17). When looking at belief as motive for action, it does not matter whether or not Crick's belief in a vitalist threat is historically supported by evidence. It is beside the point.

At the time when Crick wrote *Of Molecules and Men*, the molecular vision of life was steadily gaining prominence, with thriving research centres and a growing number of Nobel laureates. Still, its dominance over the life sciences was far from complete; and its worldview, which has been regularly denounced as the unwarranted belief that physico-chemical determinism and reductionism could address the entirety of life's phenomena,^15^ had yet to expand its realm, to developmental biology, neurobiology, neurophysiology and the other mind-brain sciences.^16^ *Of Molecules and Men* was Crick's action plan for eradicating ‘vitalism’, used as a blanket term for what he viewed as unscientific, i.e. non-deterministic and non-reductionist, belief systems. The book even outlined an educational programme, stipulating that “… all university students should be taught a subject that might be called ‘The Map of Science’”. An expected benefit would be to direct young researchers towards those areas in the “still almost virgin territory” of biology which remained in need of exploration. Even liberal arts students should be taught “one particular branch of science in rather more depth … to give them some insight into science and the scientific method.” (Crick, 1966, pp. 94–95) Had Crick had his way, he would have mass-indoctrinated the younger generations into a broadly positivist worldview.

The extract from Brenner's interview quoted earlier^17^ shows that a narrow focus at the individual level provides only limited insight into Crick's scientific evangelism. To describe how it felt to do molecular biology at the LMB in the early days, Brenner repeatedly resorts to religious metaphors^18^: “a handful of people who saw the light”; “belonging to an evangelical sect”; “preach to the heathens.” All this points to the militant character of a communal endeavour, which did not involve just Crick and Brenner but also their associates at the LMB. It was an endeavour shared by the transnational ‘new biology’ community^19^ as exemplified by Jacques Monod's *Chance and Necessity*,^20^ and Francois Jacob's *The Logic of Life*,^21^ essays that were both published in 1970 and both much praised and popular among scientists.^22^ Both attacked vitalism. Jacob retraced its roots into animism (Jacob, 1970, pp. 48, 264), while Monod dismissed it curtly as a belief that would only survive for as long as ‘mysteries’ subsist in biology and predicted that it would make its last but doomed stand on consciousness (Monod, 1970, p. 47). Monod's ‘frontiers’—the fields of biology where these mysteries subsist—coincided with the three areas identified by Crick, and his programme to conquer them largely parallelled Crick's ideas. According to Monod, the last frontier—the last bastion to fall—would be the mind-brain relationship, and especially consciousness (Monod, 1970, chapter 8). In fact, the ‘last frontier’ was how the brain was often characterised by molecular biologists, and in time many of them went on to research the nervous system and the brain,^23^ as Crick had already noted with satisfaction in *Of Molecules and Men* (Crick, 1966, p. 24). No wonder then that Crick liked Monod's book so much that he used it in a ‘sermon’ (his own word, apparently) to the Cambridge Humanists (Olby, 2009, p. 332).

Save molecular biologists, few in biology still saw vitalism was a threat, and agitating its spectre to mobilise researchers around a physicalist and reductionist worldview irritated some. One was evolutionary biologist and geneticist Theodosius Dobzhansky, as testified by his 1964 address to the American Society of Zoologists:

“… the contest of mechanism versus vitalism has been a dead issue in biology for at least half a century. To do research for invalidating vitalism is at present a height of futility. It is not unlike using heavy artillery to kill mosquitoes.” (Dobzhansky, 1964, p. 446).

Yet historian Robert Bud has shown that in England the debate between materialists and vitalists was alive and raging through the 1950s and well into the 1960s, with Crick a central figure in the fight (Bud, 2013). And Brenner reported in the late 1990s, “[w]hen, in a conversation with the mathematician, Kurt Gödel, in Princeton in 1972, I pointed out the nature and the importance of the binding site, he said: ‘This is the end of vitalism’.” (Bock & Goode, 1998, p. 109)

Meanwhile, in the life sciences, the threat of the molecular approach to so-called ‘vitalists’ who did not embrace physico-chemical determinism and reductionism, was becoming much stronger than the threat of the vitalists to the molecular biologists. In the early 1960s, many in molecular biology had no qualms about their colonizing ambitions towards developmental biology and the nervous system, as shown by a personal note from Brenner to Max Perutz, Director of the LMB, dated June 1963. The note describes his and Crick's ideas for the expansion of the lab's research activities:

“It is now widely realised that nearly all the ‘classical’ problems of molecular biology have either been solved or will be solved in the next decade. … Because of this, I have long felt that the future of molecular biology lies in the extension of research to other fields of biology, notably development and the nervous system. This is not an original thought, because as you well know, many other molecular biologists are thinking in the same way.” (Wood et al., 1988, p. x)^24^

From around the mid-1970s, when the colonizing enterprise had progressed much further, some life scientists and social scientists began to denounce materialist reductionism in biology as ideology, with the negative connotation given to the term by Marxist theory (Lewontin, 1992, Rose et al., 1984, Rose and Rose, 1976).

When examining Monod's and Jacob's books alongside *Of Molecules and Men*, and Brenner's imperialist push, vitalism appears to have been a straw man, whose convenient fuzziness made it possible to amalgamate several targets under a single umbrella. Taken together, these books have much in common with propaganda,^25^ accomplishing complex militant work not only against the authority of religion over certain subjects, but also against that of the humanities and classic biology.^26^ In scientific circles, Crick's book was much less successful than Jacob's and Monod's, but he compensated by doing what he did best, exploiting the credit and stature that his Nobel Prize had brought him to lecture far and wide on behalf of the ‘new biology’ and against the spectre of ‘vitalism’,^27^ in fact, taking on so many engagements that the intense travel and overwork took their toll on his health.^28^ On a reflexive note about the interplay between historiography and sources, my interpretation of Crick's, Jacob's and Monod's books as accomplishing militant work in favour of their positivist and reductionist worldview suggests that when historians use popular science books by scientists as primary sources, they should read them not just as science communication but as political manifestos—components in a consistent body of work produced by a specific social group.^29^

The manner in which these molecular biologists constructed vitalism as an outdated yet enduring nuisance ripe for eradication has much in common with the way movement organizers use collective action and framing to mobilise support, as described by social movement theory (Crossley, 2002, Snow et al., 1986, Tarrow, 1998). It even has the same emotional dimension (Goodwin et al., 2004, Tarrow, 1998). I captured an echo of this—an inkling of the surprising endurance of the ‘vitalist’ feud—when in 2008 I sat in on a keynote lecture given by a dyed-in-the-wool synthetic biologist to an audience of Artificial Life researchers: he finished his address saying that there was an obvious conclusion to be drawn from biologists' failure to define life and that is, that there is no such thing, “… life is just a term for poets, not scientists. There are only replicators with different degrees of complexity. P.S.: Many of you are closet vitalists.”^30^ Indeed, Brenner, interviewed by de Chadarevian well into the 2000s, still sanctioned the political nature of the molecular biology enterprise:

“I think the only thing you can really define is what some people call a program, but what I call a manifesto. What was the manifesto of molecular biology? Its manifesto was that we can explain biological behaviour through the properties of the large molecules within [cells], mainly the nucleic acids and proteins. That was the manifesto. … And basically, we haven't yet completed that program. … The manifesto is still here.” (de Chadarevian, 2009)

A remaining question is how Crick's efforts to organise neuroscience from the late 1970s onward, relate to his ‘anti-vitalist’ crusade. In Crick's as in Monod's discourse, the mind-brain, especially consciousness, was one of three areas of biology where ‘vitalism’ was supposedly still lurking and the one they expected to be conquered last. Through his self-constructed anti-vitalism, Crick was essentially promoting a reductionist, physicalist and deterministic approach to living phenomena, a distinct worldview that was still an integral part of his later framings of the mind and of consciousness, as is evident for instance in his 1994 book *The Astonishing Hypothesis*, whose opening sentence states that “ ‘You’, your joys and your sorrows, your memories and your ambitions, your sense of personal identity and free will, are in fact no more than the behaviour of a vast assembly of nerve cells and their associated molecules. As Lewis Carroll's Alice might have phrased it: ‘You're nothing but a pack of neurons'” (Crick, 1994, p. 3). Or as Crick wrote with Christof Koch, outlining the research programme that he would pursue to the end of his life: “We suggest that the time is now ripe for an attack on the neural basis of consciousness. Moreover, we believe that the problem of consciousness can, in the long run, be solved only by explanations at the neural level.” (Crick & Koch, 1990).

Having described Crick's dedication to organising ‘un-disciplined’ research fields still in their infancy and his crusade against vitalism, I will now turn to the way he deployed his skills as a cross-worlds influencer to achieve his aims.

## 3 A cross-worlds influencer at work

### 3.1 The importance of bridging fields

On May, 1998 Crick gave the Walter Heiligenberg Memorial Lecture at the UCSD ′98 Neuroscience Retreat, entitling his talk, ‘My Life in Science’. According to his handwritten notes, he planned to conclude thus:

“Finish: Two General Remarks:

1. Importance of close collaboration (e.g. Jim, Sydney, Leslie, Christof).^31^

2. How to bridge fields, e.g. protein chemistry and genetics, now: neuroscience and consciousness.”^32^

Crick's close collaborations are well known and documented (see especially, Brenner, 2001, Crick, 1988, Crick, 1994, Koch, 2012, Olby, 1974, Olby, 2009, Watson, 1968). Indeed, the collaboration with James Watson, which led to the elucidation of the double-helix structure of DNA in the spring of 1953, has acquired near-legendary status. His emphasis on bridging fields, which he puts on the same level of retrospective importance as his close collaborations, is more unexpected, and shows his appreciation of the major role it played in his career.

I have already shown that Crick had a distinct predilection for research fields that were still in their infancy. These fledgling scientific pursuits were inter-disciplinary in the literal sense of the word. Every new field he entered, be it ‘the frontier between the living and the nonliving’, the origin of life, or consciousness and the mind, raised questions that could not be tackled from within a single existing discipline. None had developed credible theoretical frameworks for addressing the questions it raised. In each case, the only way to build such frameworks would be to bring together different strands of research, many of them still in the bud. Throughout his scientific life, in England and in California, Crick built bridges not just between disciplines but also between experimentalists and theoreticians, making it possible to build broad theoretical frameworks firmly grounded in experimental work. This is a second major thread that runs through his life. It is also an exemplary display of cross-worlds influencing. What follows will highlight the eminently political nature of the work of the cross-world influencer, as the ‘bridging’ that Crick practiced involved carefully selecting the strands of research that he wished to bring together, overcoming insularity by cherry-picking young individual researchers for their openness to interdisciplinary collaboration, and acting as their protector and mentor.

### 3.2 Molecular biology and the LMB

That Crick perceived bridging fields as necessary to address the questions he was attracted to was already evident in his application for an MRC postgraduate studentship in July 1947. Explaining that he wished to enter biophysics to pursue his interest in “the division between the living and the non-living,” he wrote:

“It is clear that problems of this difficulty and complexity cannot hope to yield to any single form of attack; not only must a variety of methods belonging to any single science be employed … but also a variety of branches of sciences. … So wide, indeed is the range of scientific disciplines involved that it is bound to be impossible for anyone to achieve the degree of mastery in all of them which comes from working directly in a subject. What is required, rather, is:

(a) A general knowledge of these related sciences, sufficient to enable one to appreciate the advantages and limitations of their methods, and the significance of current work.
(b) A thorough grounding in at least one science. In my case this would of course, be physics; but I realise that this would have to be extended in the direction of physical chemistry.”^33^

If we trust his autobiographical recollections, he was aware that pitching his project in this manner could turn the liability of his lack of specialized expertise into an asset: “I took stock of my qualifications. … Only gradually did I realise that this lack of qualification could be an advantage. By the time most scientists have reached age thirty, they are trapped by their own expertise. … I, on the other hand, knew nothing, except for a basic training in somewhat old-fashioned physics and mathematics and an ability to turn my hand to new things.” (Crick, 1988, pp. 16–17)

The skills that Crick outlined in his MRC application, i.e. having enough knowledge of relevant fields of research to understand the gist of their work and methods, are akin to ‘interactional expertise’.^34^ And judging from Crick's 1947 application to the MRC, the research programmes that he had in mind to address the questions he was interested in were typical instances of ‘fractionated trading zones,’ whose coordination requires ‘interactional expertise’ (see Introduction). The bridging role Crick was putting himself up for fitted the profile of the ‘interactional expert’. He would hone its characteristic skills for the rest of his life. For instance, his strategy for approaching and eventually recruiting into new fields always included extensive reading.^35^ It was a reaching out strategy: he would not start interacting with researchers in a given field before he felt that he had mastered enough of their ‘language’ and figured out which problems mattered to them. Then he would engage them in extensive discussions, visit their labs and eventually become a participating witness of their experiments. Also characteristic was his ability to change registers depending on his audience, not only ‘talking the talk’ specific to different research fields, but also wearing the experimenter's cap when talking to theoreticians and vice-versa.^36^

The double-helix structure of the DNA, which earned Crick, James Watson and Maurice Wilkins the Nobel Prize in Physiology or Medicine in 1962, has become a popular icon that has been appropriated for various ends (for instance, de Chadarevian, 2002, chapter 6)—so famous, in fact, that it has eclipsed the fame of its authors. In the case of Crick, some have judged that even if he struck gold with DNA, it was just a ‘flash in the pan’ (Olby, 2009, p. 257). I will argue, on the contrary, that the discovery of DNA is emblematic of Crick's lifelong dedication to building bridges between fields, since as Sahotra Sarkar has pointed out, “[w]hat is remarkable about the double-helix model is that … it is a *confluent* model (standing at the confluence of four different research programs).” (Sarkar, 2005, p. 22)

Crick did not limit himself to playing the ‘interactional expert’ or to bridging fields in an ad hoc manner. He also realised the importance of shaping strong institutional bases in which the interdisciplinary collaborations that he was aiming for could take root and flourish. Historians of science have shown that laboratories can be read as embodiments of a moral economy: an ethos, the values and virtues of a community, its modes of recruitment and enculturation (see for instance, Daston, 1995, Kohler, 1994). This is how I conceive institution-building. Proposing that Crick was an institution-builder may sound surprising, since he was reputedly a man who disliked managerial positions and administrative chores.^37^ Yet there are different ways to contribute to building research institutions. One is to work, not through the exercise of direct administrative and hierarchical power, but by influencing.

In the course of his professional career, Crick was involved with two major research institutions, the LMB and the Salk Institute. The first he joined in 1949 when it was still the MRC Unit, housed at the Cavendish Laboratory and counted four researchers, himself included (he was a doctoral student there). When he left in 1976, it had developed into the ‘new LMB’ with four research divisions housed in a purpose-built building on the site of the old Addenbrooke hospital. From 1957 onwards, Crick was one of five scientists actively involved in negotiating the conditions of the move and the constitution of the new laboratory. Once the LMB had moved into its new buildings, Crick wielded not only influence, but actual decision-making power. With Brenner, he co-directed one of the four divisions (apparently leaving to Brenner the bulk of the administrative management work), and was one of the handful of individuals on the Governing Board. This part of the history of the LMB, and Crick's role in it, has been masterfully recounted by de Chadarevian and I can only point the reader to her work, which shows the importance of Crick's contribution to building-up the LMB over the first twenty-five years of its existence (de Chadarevian, 2002).

Still, I will add a couple of remarks. The first concerns an extract from the Memorandum for ‘A Laboratory of Molecular Biology’ presented late 1957 to the MRC, reproduced by de Chadarevian. This programmatic document, co-signed by Crick and Perutz, made the case for the expansion of the lab and outlined the desirable profile of a candidate to address “the division between the living and the non-living”:

“What sort of people work in Molecular Biology? Broadly two types of persons can be distinguished. The main body of workers consists of people who are specialists in one small part of the field, and who mainly see the problem from that angle. This is not very satisfactory. … In recent years there has grown up a small but increasing group of first-class workers who understand the subject as a whole, and are prepared, irrespective of their original training, to learn and to produce new techniques to solve the problem before them.”^38^

The passage shows that what Crick had outlined in his 1947 MRC application, turning the liability of his non-specialization into an asset, had by now become a cardinal virtue for the ideal LMB scientist.

My second remark is that Crick was concerned not just with institution building but also with the creation of an international network of ‘like-minded’ institutions, which strengthened the infrastructure in which his chosen research fields could prosper. This is shown clearly by the way he approached the French molecular biologists at the Institut Pasteur.

In April 1955, Crick wrote a handwritten note to “Dear Professor Monod”, to ask if he could take the opportunity of an upcoming private trip to Paris with his wife, to make his acquaintance. From there he plunged into a scientific discussion of a recent paper of Monod's, emphasizing how part of Monod's line of thinking appeared similar to ideas he himself had been working on. He concluded by saying that their travel dates were not yet fixed, and could thus be adapted to suit Monod. In short, he was organising the trip for the main purpose of getting acquainted with Monod, and was willing to set the dates accordingly. Monod agreed, and the meeting developed into plans for “a completely informal departmental seminar” to be given by Crick (in English not French, for which he apologised) to the Pasteurians in May 1955.^39^ Monod's feedback on the encounter to his friend and colleague Melvin Cohn was eloquent: he had been bowled over by Crick, who he described as probably the most brilliant person he had met since Joshua Lederberg, and that they had talked for two entire days of proteins' structure and diverse forms of ‘template hypothesis.’^40^

Crick's first-contact approach had all the characteristics of a cold call sales pitch, and there can be little doubt that his influencing skills had been nurtured by the social milieu in which he grew up—a world of ‘in trade’ middle-class entrepreneurs (Olby, 2009). Be that as it may, the interpersonal relationships that he created through his social skills^41^ would initiate the development of a strong, long-lasting and productive relationship between the LMB and the Institut Pasteur.

### 3.3 The Salk Institute

The second institution that Crick was involved with in the course of his career was the Salk Institute, and his involvement was not only very much upstream, as in the case of the LMB, but also lasted much longer—forty years in total. The Salk Institute was the brainchild of Jonas Salk, who had made his fortune and reputation from the first commercially viable inactivated polio vaccine in the 1950s. Instituted as a legal entity in December 1960, it was intended as an independent, not-for-profit, interdisciplinary research institution.^42^ Building work started in 1962 and the first laboratory opened in the spring of 1963, in the same timeframe as the new LMB. From the fall of 1961, when the Salk Institute still existed only on paper, Jonas Salk appointed Crick, Monod,^43^ Salvador Luria, Leo Szilard, followed by Warren Weaver a few months later, as the first Non-Resident Fellows. These could have been more or less honorary positions, allowing the institute to benefit from the considerable weight of the new Fellows' reputations. Instead, they were asked to help shape it by advising on the choice of candidates to appoint as Resident Fellows and more generally on its scientific governance. They took their task seriously.^44^ Crick completed two six-year terms as Non-Resident Fellow, spending as long as a month there each year around the time of the annual Fellows Meeting, usually in late winter or in spring. He stepped down in 1973, only to come back as visiting fellow in 1975–1976 before moving there permanently in 1976 as J.W. Kieckhefer Distinguished Research Professor,^45^ the first and last member of the Faculty who was not expected to run a lab.^46^ He remained actively involved in the scientific governance of the institute until his death in 2004.

Recruitment in research institutions is closely associated with the development of their research programmes. As such, it was a focus of Crick's attention and an area in which he came to exert a lot of influence, to the point that he became very much used to having his own way.^47^ My first example of Crick's influence concerns Leslie Orgel. I have already mentioned their collaboration in the late 1960s and early 1970s, when they worked together on issues connected to the origin of life, and described how Orgel went on to pursue a solid thirty year research programme in exobiology and the chemical evolution of life. Orgel joined the Salk Institute as Resident Fellow in September 1964 and remained there until his death in 2007; yet, in 1963, when he wanted to change fields and resign from his post as reader in the Department of Organic Chemistry in Cambridge, England, it was not the first institution he considered. At the time, he seriously envisioned joining the ‘new LMB’ crew, who were already making plans for an extension of the laboratory. Orgel was very interested, and his application to the Medical Research Council highlighted that his “interest in molecular biology has arisen largely through collaboration with members of the MRC group, particularly Dr. Crick.” He wished “to extend and continue that collaboration.”^48^ Perutz, Sanger, Crick and Kendrew all supported the idea; and Perutz, as Director of the LMB, was the spokesperson in the negotiations with the MRC, which started in April 1963. But over the following months the negotiations lingered, the MRC being well disposed towards the LMB's extension plans but less towards Orgel. Meanwhile, Orgel himself was receiving attractive offers from institutions in the United States, and could not put them on hold indefinitely.^49^ Thus, in November 1963, Crick, who was keen to keep Orgel and his proposed line of research in his sphere of influence, approached Jonas Salk with a request that Orgel be officially considered for a post at the Salk Institute. Crick pointed that Orgel had to make a decision by early March 1964, and suggested that he could be invited to visit in February at the same time as the Fellows' annual meeting.^50^ In her account of the early years of the Salk Institute, molecular biologist Suzanne Bourgeois has stressed Orgel's recruitment, with the specific strands of research that he brought over and developed, was to have a great impact on the future of the institute (Bourgeois, 2013, p. 121).

### 3.4 Neuroscience

In the same letter to Salk, in November 1963, Crick made a second suggestion that would have a great impact on the future development of the institute.^51^ In view of discussions around the institute's next appointments, he expressed the view that along with embryology, the higher nervous system was an important field, and since “not one of the Fellows is especially well-informed about either of these fields,” it would be good to have people talk to them on these topics on the occasion of the forthcoming Fellows Meeting in February 1964. He suggested that Roger Sperry^52^ should come to talk about the brain and added that, “In addition I should like to make a strong case for inviting David Hubel of the Harvard Medical School.”^53^ Crick was very impressed by the papers Hubel and Wiesel had recently published on the visual system of the cat and was engrossed at the time in reading in that area.^54^ The presentations were very successful, and led to a decision to expand the Salk Institute into the area of brain research. How neurobiology came to be integrated into the Salk Institute's research agenda is a complicated story, largely driven by the institute's financial difficulties, that deserves a detailed treatment in its own right. Suffice to say that the first actual appointment came only in 1970, with Roger Guillemin, who focused on the chemistry of the brain rather than its physiology, which was Crick's main interest.

When Crick joined the institute in 1976, neuroscience research there still focused exclusively on the lower levels of the brain (molecular and intra-cellular mechanisms), largely from a biochemical perspective. But by the end of the 1970s, when he became active in the neuroscience of vision, Crick had become convinced that although understanding the ‘structural implementation’ of the mind (i.e. the lower levels of the brain structure) was paramount, addressing higher cognitive functions would require an integrated approach that linked the molecular, cell, systems, and cognitive levels. This was reflected in the computational approach, strongly inspired by the ideas of David Marr,^55^ which Crick detailed in a successful grant application to the Systems Development Foundation in 1982. At the time, the computational approach was certainly not mainstream (Aicardi, 2014), and Crick set out to attract younger researchers to the Salk Institute and to nearby institutions like UCSD, who he thought were outstanding in their respective areas and also good candidates for interdisciplinary collaborations.^56^

Crick developed a solid relationship with computational neuroscientist^57^ Terrence Sejnowski, whom he met as a young postdoc in Stephen Kuffler's Department of Neurobiology at Harvard, around 1981.^58^ Crick did not take seriously “the computational types … if they didn't have a foot in biology”, but Sejnowski belonged to this rare breed of computational neuroscientists who also knew some biology.^59^ After a first attempt to recruit him in 1982, he eventually joined the Salk Institute faculty in 1988.^60^

At a meeting of the Neuroscience Research Program, in the early 1980s,^61^ Crick promoted the benefits of moving to Southern California to Charles ‘Chuck’ Stevens, already a well-established neurobiologist.^62^ Crick was trying to entice him to move to the San Diego area in the eventuality that he considered leaving Yale. In October 1985, he invited Stevens to spend a week at the Salk Institute, using the Visitor Program funded by his 1982 grant from the Systems Development Foundation.^63^ Discontented with Yale, Stevens agreed to join the Salk Institute in 1988 or 1989^64^ and moved there in 1990.^65^ Neuro-philosopher Pat Churchland, a close associate of Crick who moved to UCSD with her husband Paul Churchland in 1984,^66^ has confirmed that for Crick, getting Sejnowski and Stevens to Southern California was part of a deliberate plan:

“… he and I agreed that whatever it took, we had to get Terry here. And … of course it was a brilliant decision. This was partly because, Terry had an awesome breadth of knowledge in neuroscience as well as inventive computational ideas that were necessary to begin to make causal sense of how the brain does the things it does. Moreover, I think Terry more than anybody else in the San Diego area – on the mesa, as we like to say – was instrumental in bringing people together and getting people to work together, and then starting groups, and starting institutes, and he is just a genius at getting funding. The upshot was that a strong community developed with a productive spirit of collegiality. So, yes, getting Terry to San Diego was the best thing in the world for the Salk, not to mention for neuroscience at UCSD, at that time. Convincing Chuck Stevens to move to the Salk was Francis' next big project. And I remember Francis and I were attending a meeting near Harvard in Cambridge, and we went for a walk in the park during the lunch break. He was very direct about the plan; he said, now we have to hire Chuck Stevens at the Salk. Of course I was only an adjunct at the Salk, but I did help in whatever ways I could. And of course Chuck was a tremendous addition, both because of his brilliance as a scientist, but also because of his generous social nature, and his famously good judgment.”^67^

Recruited in 1987, Thomas Albright, whose research focuses on the visual system, thus summarized Crick's efforts:

“By the time I was here, [Francis] had an enormous influence over the direction of neuroscience at the Institute. I mean, there was … this molecular neuroscience group here, that had been here for some time. Francis didn't pay much attention to that … Francis wanted to see people working on big systems in the brain. So he pushed for the development of [our] group.”^68^

Much more could be said but I believe this is enough to give a flavour of the way Crick used appointments to influence the development of neuroscientific research at the Salk Institute. Today, the institute counts nine laboratories which together cover all the brain's different levels of biological organization.^69^ Stevens heads the Molecular Neurobiology Laboratory.^70^ Albright is Director of the Vision Center Laboratory and Conrad T. Prebys Chair in Vision Research.^71^ Sejnowski is Director of the Crick-Jacobs Center for Computational and Theoretical Biology and Francis Crick Chair of Computational Neuroscience, as well as Director of the Institute for Neural Computation at UCSD,^72^ and he leads the Salk Institute's participation in President Obama's BRAIN project, launched in 2013.^73^

But Crick's role in the establishment of a robust and multistranded neuroscience research base at the Salk Institute was not limited to influencing recruitment. He was also very generous with his ideas and kept suggesting new ones to pursue, occasionally with insistence, to the circle he had helped bring together. Ed Callaway, neuroanatomist and head of the Organization and Function of Cortical Circuits group at the Systems Biology Laboratories, pointed out that Crick “managed scientific directions … by influencing all the people and telling them his ideas, and what he thought was important, and what other people should do. He was hugely influential in that way.”^74^

Further, he helped establish a dense mesh of interdisciplinary collaborations, in the neurosciences broadly conceived, across South-Californian research institutions.

A notable example of the sort of semi-institutional gathering through which such collaborations developed was the Helmholtz Club, which ran from 1982 into the 2000s. Operating from UC Irvine, with participants from various research centres and from research fields as diverse as neuroanatomy, neurophysiology, psychophysics, computer science and engineering, the Helmholtz Club brought together scientists who shared an interest in the visual system and in higher cognitive functions that rely on visual perception, such as visual consciousness. Crick was its co-founder, patron and a core member for over twenty years. In a previous historical study, I have demonstrated his lasting influence over the club, the wide array of uses that such a gathering can serve when skilfully organized, and its role as a metaphorical greenhouse where a new research programme can take root and eventually flourish. In the case of the Helmholtz Club, these uses cut across the epistemological, social and political dimensions of science (Aicardi, 2014). Indeed, I would venture that this particular semi-institutional gathering played a key role in three huge and apparently inevitable developments: the framing of current ‘big brain’ projects like the American BRAIN Initiative and the European Human Brain Project within the paradigm of computational neuroscience, the key role of neural networks simulation platforms in current research, and the vision of neuromorphic hardware as a privileged physical support for brain models.

## 4 Concluding remarks

The institutional edifice of science relies explicitly on standards of evaluation that are mostly limited to citation counts, publication records and individual awards. Yet ethnographies of science over three decades have taught us that doing science day-in day-out is a highly social and collective pursuit, that it can take place in a diverse array of spaces and can involve all sorts of activities.^75^ In many scientists' memories, such activities are not central to, or even a part at all, of ‘doing science’; they hardly ever emphasize these aspects of their work, in contrast to their research interests and results, far more important to them. Still, questioning memory work, and thus questioning how central to doing science these supposedly peripheral activities actually are, is part of the historian's job.^76^ This, in turn, has important implications for notions of ‘success’ and ‘failure’ in science, as questioning the scope, and relative weight, of the activities that count towards ‘doing science’ challenges the accepted institutional standards of evaluation.

The received wisdom about Crick's long engagement with the neurosciences is that he did not make any significant breakthrough on the major problems he set out to solve and should thus be counted a failure. In the present essay, I have argued that cross-worlds influencing was part of ‘doing science’ and in this light, I have shown that Crick's contributions to the neurosciences may have been severely underestimated, and merit serious scholarly investigation. Approaching Crick as a ‘cross-worlds influencer’ makes it possible to view his diverse career as a coherent whole, giving new, politically-charged significance to a course of actions that could otherwise seem inconsistent and dispersed. It also shows how valuable the concept of cross-worlds influencer can be for pursuing the political dimension of ‘doing science’. In particular, it puts more weight on individual agency than the concepts of ‘go-between’, ‘broker’, or ‘bridge scientist’^77^, which all emphasize the intermediary role that these figures play but underplay their role in steering and shaping scientific developments.

To conclude, I would like to address an issue that I have left hanging, namely the question of why it matters to relate Crick's activities in his various fields of research to his life-defining ‘anti-vitalism’. It matters first from a historiographical perspective, because it gives a new and consistent perspective on Crick's diverse career, which, if I follow Thomas Hankins' defence of biography in history of science, should be a major aim for scientific biography (Hankins, 1979, p. 8). But more importantly, it matters because Crick's self-constructed anti-vitalism encapsulates the specific worldview that he carried across fields. This, in turn, motivated him to influence the conditions under which interdisciplinary collaborations could take shape, privileging the possibilities of certain interdisciplinary hybrids which he saw as congruent with his ‘anti-vitalism,’ while rejecting others. One example, in the early days of the Salk Institute, was Crick's opposition to Warren Weaver's proposal to have an evolutionist like Ernst Mayr or George Gaylord Simpson join as a Resident Fellow, which he justified on the grounds that an evolutionist biologist would simply not fit with the existing Resident Fellows, and not see ‘eye to eye’ with (some of … ) the Non-Resident fellows (Olby, 2009, p. 373).

In summary, the present study, together with my previous study of the Helmholtz Club and Crick's role in it (Aicardi, 2014), shows how historians can use cross-worlds influencers as guides into the thickets of contemporary big bioscience. By following Crick in this role, one can navigate semi-institutional networks that cut across many research fields, funding and policymaking bodies, and institutions. Like one of García-Sancho's administrators, Crick played an influential role (if self-imposed and self-constructed when theirs was part of the job description) brokering and establishing trust between distinct research communities and building-up institutions. I would like to propose that more generally, ‘following the cross-worlds influencers’ may be a fruitful heuristic for historians probing the rhizomic and genealogic entanglements of modern big bioscience.

## Footnotes

1 In the UK, Big Data is a cross-cutting ‘vital part’ of RCUK strategy: http://www.rcuk.ac.uk/research/infrastructure/big-data/. For the Arts and Humanities Research Council (AHRC), the Big Data research programme is at the core of ‘Digital Transformations’, one of the four AHRC strategic themes for 2013–2018: http://www.ahrc.ac.uk/Funding-Opportunities/Research-funding/Themes/Digital-Transformations/Pages/Big-Data.aspx. Links were last consulted 12/03/2015.

2 See for instance: Cialdini, 2007, Grenny et al., 2013. http://www.forbes.com/2011/01/03/influence-persuasion-cooperation-leadership-managing-ccl.html; https://hbr.org/2008/02/exerting-influence-without-aut.html; http://carlsonschool.umn.edu/executive-education/programs/power-and-influence. All links last consulted 13/03/2015.

3 See for instance Manning and Robertson, 2003, Johnson-Cartee and Copeland, 2004, Hoy and Smith, 2007, Manning et al., 2008a, Manning et al., 2008b, Manning et al., 2008c, Kaufman, 2011.

4 See in particular, Collins and Evans, 2002, Collins et al., 2007, Collins, 2011.

5 Even though Collins et al. (2007) point at gravitational waves research as a typical example, understandably considering the number of years that Collins has been involved with this field, the authors point that “… when examined closely, what appear to be integrated networks of scientists are really conglomerations of small groups bound together by rich interactional expertises.” (Collins et al., 2007, p. 661) The domain of life sciences has proven an excellent candidate for the development of such ‘big science’ projects, due to complexity of the subject matter.

6 I have borrowed and translated the notion of ‘semi-institutional’ from social geographer Hester Parr, who has used it in her work on psychiatric-medical care in Parr (2000) to describe sites of community care which are neither institutional locations nor free from clinical associations, in-between places “influenced partially through grass-roots visions of community inclusion and support, and partially through state regulation and funding.” In Ilie (2001), linguist Cornelia Ilie has used the term ‘semi-institutional’ in a related manner, to characterise discourse that “exhibit[s] a mixture of characteristics pertaining to both casual conversation and institutional discourse.”

7 Beside the present paper, see Aicardi (2014).

8 For the close collaboration between Sydney Brenner and Crick, which lasted from 1957 until 1976, see de Chadarevian, 2002, Olby, 2009.

9 Website of the MRC Laboratory of Molecular Biology: http://www2.mrc-lmb.cam.ac.uk/group-leaders/, last consulted 03 May 2015.

10 Proof that Crick did not take their effort for anything more than speculative, he sent an advanced draft of their joint paper on “Directed Panspermia” to Jacques Monod at the Institut Pasteur, writing in the accompanying letter: “As you can see, it could be classed as Higher Science Fiction.” (Institut Pasteur, Monod archive, Box MON.Cor.04, Folder ‘Francis Crick’, letter from Crick to Monod, 1st November 1972).

11 The hypothesis that life on earth may have been seeded by microorganisms originating from other planets and transported through space.

12 Wellcome Library, Francis Crick Papers, folder PP/CRI/D/1/5/14, letter Ramachandran to Crick, 8 June 1982.

13 This view was widely shared by the scientists from diverse neuroscientific and neuroengineering fields that I interviewed at the Salk Institute and Caltech in the Autumn of 2012.

14 This extract has been transcribed from the recording kindly provided to me in the Spring of 2012 by Annette Faux, the LMB archivist.

15 This critique has come not only from theorists of complexity (see for instance, Amzallag, 2002, Morin, 1990, Prigogine and Stengers, 1979) but also from number of biologists who have had serious reservations about the deterministic and reductionist project of molecular biology (see Lewontin, 1992, Rose et al., 1984, Rose and Rose, 1976).

16 The 1960s saw molecular biologists turning to developmental biology with ambitious research programmes, like that hatched by Brenner in 1963 through his conversations with Crick around *Caenorhabditis Elegans* (de Chadarevian, 1998). As for the neurosciences, the term ‘neuroscience’ was only coined around 1962 (Rose & Abi-Rached, 2013), and computational, systems as well as cognitive neuroscience had still to come into existence.

17 Extract from Dzeng (2014).

18 Although it falls squarely outside the remit of the present paper, I think that the ‘secularly religious’ register of scientific discourse in 20th and 21st century life sciences deserves more attention. For instance, see recent posting on The New York Times website: http://www.nytimes.com/2015/03/15/opinion/sunday/the-church-of-ted.html?_r=1, consulted 15/03/2015.

19 The ‘new biology’ was amply nurtured on both sides of the Atlantic by the Rockefeller Foundation under the influence of Warren Weaver, from the 1930s onward—but that's another story (see in particular Abir-Am, 1987, de Chadarevian, 2002, Gaudillière, 2002, Kay, 1993).

20 Which subtitle is *Essay on the natural philosophy of modern biology*.

21 Which subtitle is *A history of heredity*.

22 Monod and Jacob were both Nobel laureates and leading researchers at the Pasteur Institute in Paris, a well-known stronghold of molecular biology.

23 See for instance Brenner (2001), pp. 120–121.

24 Wood et al. (1988), pp. ix–xii: The source for Brenner's memorandum is a commemoration book that was edited by ‘the Community of *C. elegans* Researchers’ over two decades after the launch of the research programme, to celebrate its outstanding success and “… be the sourcebook of the worm for some time to come.” Brenner reproduced it alongside excerpts from the October 1963 proposal to the MRC, in annex to his Foreword to the volume. As commemoration work, it fits well with Abir-Am's historical analysis of the socio-political dimension of commemorative practices in molecular biology, in Abir-Am (1999).

25 For the present purpose, a usefully synthetic definition of propaganda, and its distinction from persuasion, is provided by Johnson-Cartee & Copeland (2004), p. 3: “Persuasion utilizes messages that tell individuals to adopt a new belief or attitude, or to engage in a new behaviour for their own personal benefit. … Propaganda, on the other hand, utilizes messages that tell individuals the attitudes, beliefs, and behaviours found desirable in their social groups.”

26 On a reflexive note about the interplay between historiography and sources, my interpretation of Crick's, Jacob's and Monod's books as accomplishing militant work in favour of their positivist and reductionist ideology confirms that as primary sources, popular science books written by scientists can be read as more than just science communication but as political manifestos, and as more than itemized pieces but as part of a consistent body of work produced at the level of a social group.

27 In Olby (2009), p. 240, Olby argues that Crick's renown as a skilled lecturer led to a great number of invitations to give talks, and that for a time he became something of a “traveling speaker; he played the role of molecular evangelist for the new science.”

28 In 1971 he had to wind down on doctor's orders and get some rest, as he explained to Monod in a letter (Institut Pasteur, Monod archive, Box MON.Cor.04, Folder ‘Francis Crick’, letter from Crick to Monod, 29 November 1972).

29 The case of *Of Molecules and Men* shows that disregarded popular science pieces can bring insights for understanding scientists' lives that their professional papers would not necessarily yield.

30 Keynote lecture given on 7 August 2008 at ALIFE XI conference (Chichester, UK).

31 The first names refer to James Watson, Sydney Brenner, Leslie Orgel and Christof Koch.

32 Wellcome Library, Francis Crick Papers, folder PP/CRI/L/7/8.

33 National Archives, MRC papers, staff file FD 21/13 Crick, DR F.H.C., ‘Application for MRC Studentship’, attached with Crick's CV to memorandum by Edward Mellanby dated July 7, 1947.

34 The importance of brokerage for bottom up interdisciplinary projects has also been recognised from a science policy studies perspective, like in Corley, Boardman, & Bozeman (2006).

35 His extensive reading has been widely remarked upon by his ex-colleagues and collaborators that I have interviewed, and is also a trait of his personality that Olby has put forward in his biography of Crick, in Olby (2009).

36 This is for instance apparent when contrasting the empirical chapter he co-authored for the largely theoretical *Parallel Development Processing*. *Explorations in the Microstructure of Cognition*, vol. 1: “Foundations” (McClelland & Rumelhart, 1986), with the theoretical “Framework for Consciousness” that he co-authored with Christof Koch (Crick and Koch (2003)).

37 This is well documented in Olby (2009), and has been unanimously confirmed by those who worked with him.

38 LMB Archive, File ‘Laboratory of Molecular Biology, Establishment of—Memorandum to MRC’. See also de Chadarevian (2002), chapter 7.

39 Pasteur Institute Archive, Jacques Monod Archive, Boxes MON.Cor.04, Folder ‘Crick, Francis’.

40 “Nous avons eu, il y a quinze jours, la visite de Francis Crick qui m'a complètement séduit. C'est probablement le garçon le plus brillant que j'ai rencontré depuis Josh–Lederberg. Nous avons parlé deux jours de suite de structure de protéines et de diverses formes de ‘template hypothesis’. ” (Pasteur Institute Archive, Jacques Monod Archive, Boxes MON.Cor.03, Folder ‘Cohn, Melvin’).

41 Crick's approach has many similarities with the brokering between ‘trust networks’ that has been described by political sociologist Charles Tilly, in Tilly (2005).

42 Salk's initial plan was to push interdisciplinarity beyond the ‘two cultures’ divide and bring together the life sciences and humanities, a plan that did not survive the 1970s. On the humanities programme at the Salk Institute, see Aronova (2012).

43 My personal research into the individual records of Crick and Monod (Pasteur Institute Archive, Jacques Monod Papers, ‘Relations of Jacques Monod with the Salk Institute’ (Boxes MON.Ins.05 and MON.Ins.06); Wellcome Library, Francis Crick Papers, ‘Salk Institute for Biological Studies 1953–2004’ (Series PP/CRI/C)) has led me to conclude that this joint appointment did much to tighten the connection between the LMB and Institut Pasteur in various ways, and expand it into California.

44 This assessment is based on my personal research into the Salk Institute files in Jonas Salk papers, as well as into the individual records of Crick and Monod: UCSD Library, Mandeville Special Collections, Jonas Salk papers, Salk Institute Files (MSS1 Series 6); Pasteur Institute Archive, Jacques Monod Archive, ‘Relations of Jacques Monod with the Salk Institute’ (Boxes MON.Ins.05 and MON.Ins.06); Wellcome Library, Francis Crick Papers, ‘Salk Institute for Biological Studies 1953–2004’ (Series PP/CRI/C).

45 Interestingly, Crick's correspondence regarding the negotiations of the Kieckhefer professorship were in his private papers, not his professional papers, which is evidence that sensitive professional correspondence will not necessarily end up in a scientist's professional papers (UCSD Library, Mandeville Special Collections, MSS 660, Box 13, folders 10).

46 See Olby (2009); it has been confirmed by long-standing members of Faculty at the Salk Institute.

47 This was confirmed by several of the still active scientists that I have interviewed at the Salk Institute in the Autumn of 2012. Some emphasized the point by using as counterexample the one memorable time in the early 1990s when he didn't succeed in getting a particular neuroanatomist hired (Thomas Albright, personal communication, 02/10/2012; Charles Stevens, personal communication, 26/09/2012).

48 LMB Archives, File ‘1st extension’, document ‘Experimental Approaches to the Problem of Biogenesis by Leslie Orgel’.

49 The correspondence between the LMB and the MRC, memorandums of the MRC, internal memorandums of the LMB, and the various stages of the proposal, are in LMB Archives, File ‘1st extension’.

50 Wellcome Library, Francis Crick Papers, PP/CRI/C/2/1/1, letter from Francis Crick to Jonas Salk, November 25, 1963.

51 Again confirmed by Bourgeois, in Bourgeois (2013), p. 121.

52 Researcher in neuropsychology and neurobiology, famous for his split-brain experimental work.

53 Wellcome Library, Francis Crick Papers, PP/CRI/C/2/1/1, letter from Francis Crick to Jonas Salk, November 25, 1963.

54 See for instance Brenner's testimony, in Brenner (2001). Hubel's and Wiesel's research on the visual system of the cat won them the Nobel Prize in 1981, jointly with Sperry.

55 Marr's ideas were about to be published posthumously in his book *Vision*, which was to become one of the landmarks of computational neuroscience (see Marr, 1982).

56 See quote from Ramachandran (2004) in Section 2: “… he was instrumental in having many of us in neuroscience and psychology move to La Jolla in the early eighties—transforming it into “neuron valley” (He had the foresight to bring Terry Sejnowsky and the Churchlands to UCSD at a time when the kinds of topics they worked on were not considered especially fashionable). We all thought of him as a great Sequoia tree under whose branches many of us saplings eked out a precarious living.” Also quote from Siegel & Callaway (2004), in Section 3: Crick was an “evangelical atheist” who “… was building an army to help him take on consciousness.” For more on Crick's plans for neuroscience, see Aicardi (2014).

57 The term ‘Computational neuroscience’ had not yet been coined, it would be a few years later only around 1985 (see Aicardi, 2014).

58 Kuffler had set up at Harvard one of the very first Departments of Neurobiology, in 1966.

59 Thomas Albright, personal communication, 02/10/2012.

60 Wellcome Library, Francis Crick Papers, under PP/CRI/D and PP/CRI/J: ‘Correspondence: S’ folders from 1978 to 1981 to 2002–2003.

61 Crick had become became an Active Associate of the NRP in the late 1970s, and remained an involved and regular attendant of its Stated Meetings even after his transfer to Honorary Associate status in 1989 at the end of his term, until 2003 (Wellcome Library, Francis Crick Papers (PP/CRI): there are many folders related to Crick's involvement with the NRP, containing some correspondence as well as programmes for the Stated Meetings of the NRP Associates that he attended regularly from the time he became an Active Associate in 1978 until the 2000s.) The NRP was typical of the kind of interdisciplinary semi-institutional gathering that Crick favoured for the potential it offered to his ‘influencing’ activities.

62 Stevens has made especially notable contributions to understanding the molecular basis of synaptic transmission.

63 For more on the SDF grant, see Aicardi (2014): it was also used to bankroll the Helmholtz Club in its early years.

64 When I interviewed him he did not remember the exact year.

65 Charles Stevens, personal communication, 26/09/2012.

66 They are known in particular for their ideas of ‘eliminative materialism.’

67 Pat Churchland, personal communication, 05/10/2012.

68 Thomas Albright, personal communication, 02/10/2012.

69 The Salk Institute laboratories are listed at http://www.salk.edu/faculty/laboratories.html, consulted 09/11/2014.

70 http://www.salk.edu/faculty/stevens.html, last consulted 18/06/2015.

71 http://www.salk.edu/faculty/albright.html, last consulted 18/06/2015.

72 http://inc.ucsd.edu/people.html, last consulted 04/05/2015.

73 InsideSalk newsletter, August 2013, ‘Neuroscientist Terry Sejnowksi attends White House announcement of collaborative BRAIN Initiative,’ http://www.salk.edu/insidesalk/articlenin.php?id=429, consulted 09/11/2014.

74 Ed Callaway, personal communication, 01/10/2012.

75 Here is a subjective selection among the very many references available, covering a wide range of topics and styles: Latour and Woolgar, 1986, Traweek, 1988, Helmreich, 1998, Collins, 2004, Dumit, 2004.

76 For discussions in historiography of contemporary science and technology about the conflicts that may arise between retrospective memories and documents written at the time of the events taking place, see Gaudillière (1997) and Hoddeson (2006).

77 The concept of the ‘bridge scientist’ is developed in Anbar (1986).
